# Data Mining Methods for Omics and Knowledge of Crude Medicinal Plants toward Big Data Biology

**DOI:** 10.5936/csbj.201301010

**Published:** 2013-03-23

**Authors:** Farit M. Afendi, Naoaki Ono, Yukiko Nakamura, Kensuke Nakamura, Latifah K. Darusman, Nelson Kibinge, Aki Hirai Morita, Ken Tanaka, Hisayuki Horai, Md. Altaf-Ul-Amin, Shigehiko Kanaya

**Affiliations:** a Graduate School of Information Science, Nara Institute of Science and Technology, Nara 630-0101, Ikoma, Japan; b Department of Statistics, Bogor Agricultural University, Jln. Meranti, Kampus IPB Darmaga, Bogor 16680, Indonesia; c Biopharmaca Research Center, Bogor Agricultural University, Kampas IPB Taman Kencana, Jln. Taman Kencana No. 3 Bogor 16151, Indonesia; d Maebashi Institute of technology, 450-1 Kamisadori, Maebashi-shi, Gunma, 371-0816 Japan; e Department of Medicinal Resources, Institute of Natural Medicine, University of Toyama, 2630 Toyama, 930-0194, Japan; f Department of Electronic and Computer Engineering, Ibaraki National College of Technology, 866 Nakane, Hitachinaka, Ibaraki 312-8508, Japan

## Abstract

Molecular biological data has rapidly increased with the recent progress of the Omics fields, e.g., genomics, transcriptomics, proteomics and metabolomics that necessitates the development of databases and methods for efficient storage, retrieval, integration and analysis of massive data. The present study reviews the usage of KNApSAcK Family DB in metabolomics and related area, discusses several statistical methods for handling multivariate data and shows their application on Indonesian blended herbal medicines (Jamu) as a case study. Exploration using Biplot reveals many plants are rarely utilized while some plants are highly utilized toward specific efficacy. Furthermore, the ingredients of Jamu formulas are modeled using Partial Least Squares Discriminant Analysis (PLS-DA) in order to predict their efficacy. The plants used in each Jamu medicine served as the predictors, whereas the efficacy of each Jamu provided the responses. This model produces 71.6% correct classification in predicting efficacy. Permutation test then is used to determine plants that serve as main ingredients in Jamu formula by evaluating the significance of the PLS-DA coefficients. Next, in order to explain the role of plants that serve as main ingredients in Jamu medicines, information of pharmacological activity of the plants is added to the predictor block. Then N-PLS-DA model, multiway version of PLS-DA, is utilized to handle the three-dimensional array of the predictor block. The resulting N-PLS-DA model reveals that the effects of some pharmacological activities are specific for certain efficacy and the other activities are diverse toward many efficacies. Mathematical modeling introduced in the present study can be utilized in global analysis of big data targeting to reveal the underlying biology.

## 1. Introduction

Data-intensive sciences have progressed in modern astronomy [1], biology [2–8], computational materials science [9], ecology [10, 11] and social science [12] because open-access data has increased drastically. Data-intensive or -driven discovery in biology requires a large open pool of data across the full breadth of the life sciences and the access to the pool will invite “New” logic, strategies and tools to discover new trends, associations, discontinuities, and exceptions that reveal aspects of the underlying biology [2, 5, 6]. Big data biology, which is a discipline of data-intensive science, was proposed based on the rapid increasing of omics data produced by genomics, transcriptomics, proteomics and metabolomics [2–8]. This situation is also a feature of the ethnomedicinal survey and the number of medicinal plants is estimated to be 40,000 to 70,000 around the world [13] and many countries utilize these plants as blended herbal medicines, e.g., China (traditional Chinese medicine), Japan (Kampo medicine), India (Ayruveda, Siddha and Unani) and Indonesia (Jamu). Blended herbal medicines as well as single herb medicines include a large number of constituent substances which exert effects on human physiology through a variety of biological pathways. To comprehensively understand the medicinal usage of plants based upon traditional and modern knowledge, we add to KNApSAcK Family database systems the selected herbal ingredients i.e., the formulas of Kampo and Jamu, omics information in plants and humans, and physiological activities in humans [14–16]. These information need to be connected in a way that enables scientists to make predictions based on general principles.

In this mini-review, we discuss the usage of KNApSAcK Family DB in metabolomics, explain mining techniques such as principal component analysis (PCA), partial least square regression (PLSR) and multiway model, and show their application on Indonesian blended herbal medicines (Jamu) as a case study.

## 2. KNApSAcK Family Database

**Figure 1:**
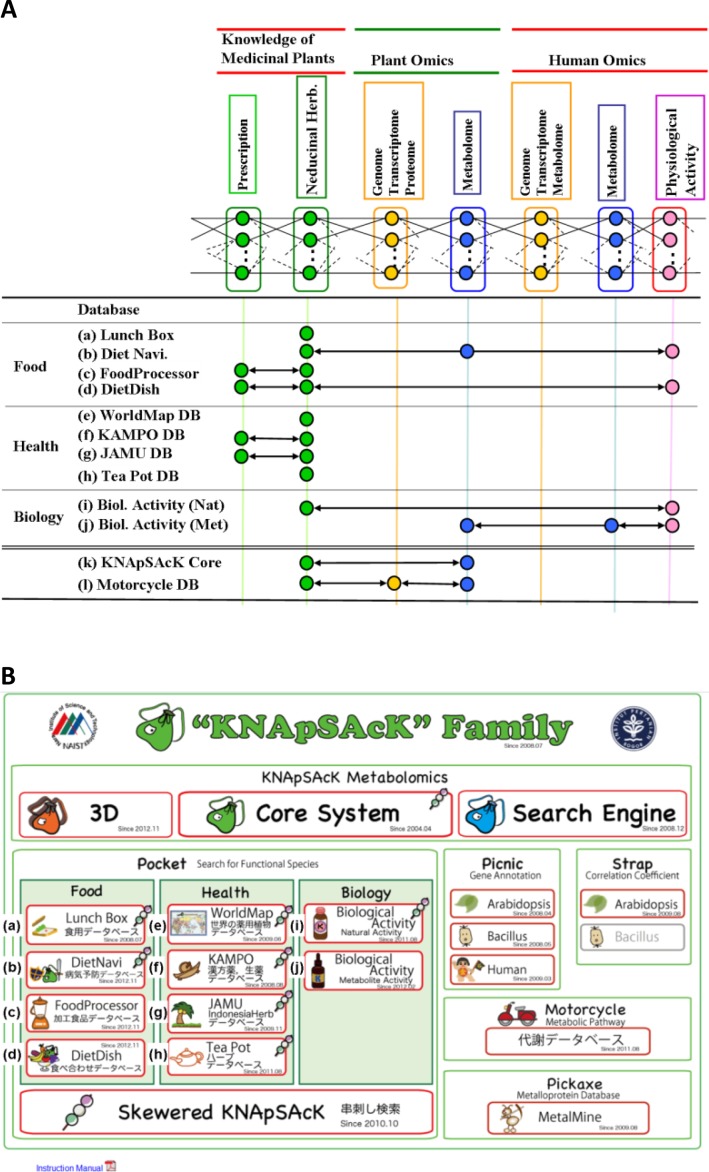
Integrated platform of knowledge of medicinal plants and plant and human –omics and KNApSacK Family databases. **(A)** The relations of attributes among individual DBs. **(B)** Main window of KNApSAcK Family DB, indexes from a to i in panel A correspond to those in panel B.

Omics biology, like most scientific disciplines, is in an era of accelerated increase of data, so called big data biology [2–8]. Large-scale sequencing centers, high-throughput analytical facilities and individual laboratories produce vast amounts of data such as nucleotide and protein sequences, gene expression measurements, protein and genetic interactions, mass spectra of metabolites and phenotype studies. The goal of investigating the interactions between medicinal/edible plants and humans is to comprehensively understand the molecular mechanism of medicinal plants on human physiology based on current and traditional knowledge. Optimization of blended herbal formulas should be developing using information derived from plant and human omics. To reach this goal we need to develop databases based on the platform shown in Fig. 1A. KNApSAcK family DBs have been developed for this purpose [14–16]. Relations among individual DBs are illustrated in Fig. 1A and main page of KNApSAcK Family DB is shown in Fig. 1B.

Four DBs (Lunch Box DB, DietNavi DB, Food Processor DB and DietDish DB, a-d in Fig. 1) are about Food & Health related with Japanese foods and ingredients explained in Japanese language because initially we developed them targeting the Japanese people, but we are planning to translate them into English as early as possible. Lunch Box DB comprises information on 800 edible species which include the species introduced to Japan from outside or originally grown in Japan, general information of the crops and the effect of them on human health.

Noncommunicable diseases such as heart disease, metabolic disease, cancer and respiratory disease, which superseded the infectious diseases because of the development and widespread distribution of vaccines and antimicrobial drugs, account for 60% of all deaths worldwide and 80% of deaths in low- and middle-income countries [17]. Food and ingredients in sanative diet and more effective combination of foods beneficial against those noncommunicable diseases are accumulated in DietNavi and DietDish DBs, respectively (b and d in Fig. 1). FoodProcessor DB comprises 309 retortable pouch foods encompassed by 261 food ingredients produced in Japan, and connected with DietNavi and KNApSAcK core by species names of foods.

To systematize crude drugs by multifaceted view points, we have developed four DBs (WorldMap, KAMPO, JAMU and TeaPot DBs as shown in e-h of Fig. 1). The KNApSAcK WorldMap DB comprises 46,256 geographic zone-plant pair entries in 217 geographical zones except mini-states such as the Principalities of Liechtenstein, Monaco and Andorra, and the Vatican City. Prescriptions corresponding to Japanese and Indonesian herbal medicines have been accumulated in KAMPO and JAMU DBs, respectively. KAMPO DB is comprised of 1,581 primary formulas classified in to 336 formula names encompassed by 278 medicinal plants which are approved by the National health insurance authority in Japan. JAMU DB is comprised of 5,310 formulas encompassed by 550 medicinal plants and 12 anatomical regions which are approved by the National Agency of Drug and Food Control (NA-DFC) of Indonesia. Medicinal/edible plants reported in the scientific literature have been classified into geographic zones using the International Organization for Standardization (ISO3166), which defines geographic zones based on the borders between nations and small islands. Herbs are defined as any plants with leaves, seeds, and flowers used for flavoring, food, medicine, perfume and parts of such a plant as used in cooking. Those are accumulated in TeaPot DB.

Two types of biological activities, that is, activities of natural resources and metabolites to other species including human, i.e., antibiotic, anticancer and so on are accumulated in Natural Activity and Metabolite Activity DBs (Fig. 1B), respectively. The former and the latter comprised 33,703 and 6,677 entries, respectively. For extension of species-metabolite relationship DB to metabolic pathways, it is needed to design secondary metabolic pathway DB for detection of metabolic pathways based on enzyme reactions and prediction of reactions by peptide sequences. So we have developed Motoercycle DB containing 2,421 entries. The metabolomics of plants is developing rapidly [18-20 and references in Table 1], and it will be an important topic in the systems-biological studies of interactions between plants and humans, which is included in the topics of big data biology [2–8], with the goal of achieving a holistic understanding of plant function and healthcare, including the activity of medicinal plants as well as interaction between plants and their environment [14–16, 21, 22].

To facilitate access to metabolite information obtained from analytical techniques, we have developed species-metabolite relationship DB (KNApSAcK Core DB) which contains 106,418 species-metabolite relationships encompassing 21,705 species and 50,897 metabolites. Nine databases of KNApSAcK family (except DietDish) are connected with KNApSAcK Core DB to easily obtain candidates of secondary metabolites in species utilized in several purposes [23]. The KNApSAcK Core DB was utilized in very diverged purposes of metabolomics studies including identification of metabolites (‘Exp’ in Table 1), construction of integrated databases (‘DB’), bioinformatics and systems biology (‘Bioinfo’), and cited in at least 110 papers listed in Table 1, that is, in 29 papers in the period of 2006-2008, 25 papers in the period of 2009, 20 papers in 2010, 18 papers in 2011, 18 papers in 2012-2013. In addition, it was applied in diverged species from bacteria to plants and animals, in total 28 species, that is, *Angelica acutiloba* [74], *Arabidopsis lyrata* ssp. *petraea* [56], *Arabidopsis thaliana* [25, 30, 33, 35, 37, 46, 47, 62, 70, 86, 99, 103, 104, 108, 109, 121, 122], *Atriplex halimus* [127], *Bacillus subtilis* [113], *Brassica oleraceae* var *capitata* [60], *Brufelsia calycina* [81] *, Capsicum sp*. [123] *, Citrus sinensis* [131], *Curcuma longa* [77], *Ephedra* sp. [67], *Escherichia coli* [51], *Fragaria* x *ananassa* [40, 43, 44], *Fragaria vesca* [105], *Glycine max* [53], *Glycyrrhiza uralensis* [94] *, Hordeum vulgare* [80, 102], *Homo sapiens* [63, 101], *Jatropha curcas* [124, 125], *Malx* x *domestica* [126], *Ophiorrhiza pumila* [117], *Oryza sativa* [49, 61], *Papaver somniferum* [42], *Rattus norvegicus* [39, 97], *Rizotania solani* [79] *, Solanum lycopersicum* [45, 48], *Solanum tuberosum* [98] and *Zea mays* [120].

**Table 1:** Studies that cite KNApSAcK Core DB.

Article type	The purpose of study [References]
< 2006-2008 >	
**Review**	Bridge between Chemistry and Biology [24], GC-MS DB [29], Metabolomics technologies [31], Functional genomics research strategy of combining transcriptome and metabolome [32], The role of MS in metabolomics [34], Mass spectrometry platforms [38], Metabolomics technologies and functional genomics platform [42], Technology and informatics [49], Atmospheric pressure ionization mass spectrometry [52]
**Exp**	Metabolite accumulation caused by herbicidal enzyme inhibitors [30], Assignment of UGT89C1 to a flavonol 7-O-rhamnosyltransferase [33], Light/dark regulation of metabolite activities [35], Characterization of mutants in flavonoid and phenylpropanoid biosynthetic pathways [37], Metabolism of dietary phytochemicals [39], Metabolic networks in primary and secondary pathways for achene and receptacle [40], High-resolution mass spectrometry and 13C-isotope labeling of entire metabolomes [41], Phenolic biosynthesis pathway [43], Metabolic profiling in strawberry receptacle development [44], Regulation of glucosinolate biosynthesis [46], Integrated analysis of metabolome and transcriptome [48], Protocol in metabolite fingerprints [50]
**Bioinfo**	Metabolome platform DrDMASS in FT-ICR-MS [25], Taxonomic diversity of flavonoids [26], MS Peak storage and processing [28], Metabolite annotation based on MS and MS2 [45], Identification of metabolites by MS and MS-tagged MS2 data [47], Metabolome platform DrDMASS in FT-ICR-MS [51]
**DB**	Chemical biology [27], Metabolome tools and databases [36]

< 2009 >	
**Review**	Integrated omics [58], MS-based technologies [59], Web-resources in MS-based metabolomics [75], Functional genomics [78]
**Exp**	Metabolic profiling in cold-temperature [56], Antioxidant compounds in white cabbage during winter storage [60], Hydroxylation of fatty acids by P450 proteins [62], Dietary phytochemicals and human [63], Classification of Ephedra sp. [67], Selection of metabolites [68], Matrix-assisted laser desorption/ionization mass spectrometry [69], Determination of gene function [70], Quality assessment [73, 74], Diarylheptanoid biosynthesis [77]
**Bioinfo**	Annotation of metabolite information to MS [53], Tools for the annotation of High Resolution MS metabolomics data [57], Comparison of metabolite DB using rice metabolites [61], Assessment of annotation of metabolites using FDR [64], Graph representation of multiple databases [65], Peak detection based on MS/MS patterns [66], Complexity of relation between plants and metabolites [71], Metabolic pathway prediction [72], Metabolite Complexity of relation between plants and metabolites [71], Metabolic pathway prediction [72], Metabolite annotation [76]
**DB**	Embedded string-search commands on MediaWiki [54]

< 2010 >	
**Review**	MS data processing [84], Metabolomics in plant ecology and genetics [85], Identification of metabolites [87], FT-ICR-MS, Reaction representation based on van Krevelen diagram [89], Relationship among individual omics data based on multivariate analysis and DB [16], Dietary intake [90], Functional Genomics [92], Annotation of gene function based on co-response gene and identification of metabolites [95]
**Exp**	Metabolite composition [79], QTL of barley, against Fusarium head blight [80], Changing color of flower from dark purple to white [81], Metabolic profiling of different tissues [86], Quality assessment [94]
**Bioinfo**	Chemical similarity search and substructure matching of compounds [82], Multiple metabolomics platforms for different types of MS [91], MS data processing [96], Network analysis of species-metabolite relations [97]
**DB**	MassBank, MS DB [83], Polyphenol contents in foods [88], Binzylsioquinone alkaloids [93]

< 2011 >	
**Review**	Pesticide research [100] ****, Metabolome DB [108] ****, Traditional medicinal plants [111] ****, Pesticide research [113]
**Exp**	Hepatotoxicity [55], Subcellular distribution of metabolites [99], Assessment of metabolites of barley against Fusarium head blight [102], Metabolic responses of ultraviolet-B light [103], Transport of 12-Oxo-phytodienoic acid-glutathione into vacuole [104], Demetylation of oligogalacturonides by FAPE1 leads to defense against fungus Botytis cinerea [105], Cytochrome P450, CYP81F4 [109], Imaging mass spectrometry [112]
**Bioinfo**	QTL informatics [98], Metabolomics in medical purpose with systems chemical biology and chemoinformatics [101], Molecular formula annotation of polar and lipophilic metabolites [107], Metabolic profiling [114]
**DB**	Food phytochemicals [106], Medicinal plants in Indonesia [110]

< 2012-13 >	
**Review**	Plant responses to abiotic stress [115], Phytoalexins [118], Plant biotechnology [119], Integrative system biology [121], Systems biology in Japanese traditional Kampo medicine [15]
**Exp**	Camptothecin biosynthesis [117], Herbivore (*Spodoptera littoralis*)-induced metabolites [120], Natural distance [122], Molecular marker [123], Metabolic changes during fruit maturation [124], Metabolites in seed kernels [125], mQLT [126], Salt and drought stress [127], Mass spectrometric imaging [130], Defence against pathogens (Penicillium digitatum) [131]
**Bioinfo**	Repository for metabolomics studies [128], Visualization of metabolome data [129]
**DB**	Metabolite annotation [116]

In the period of 2006-2008, many review papers [‘Review’ in Table 1] focused on metabolomics platforms integrated by mass-spectrometry and metabolite databases including KNApSAcK Core [29, 31, 34, 38, 42, 49, 52] and on linking chemistry with biology [24], and on metabolome researches targeting the model plant *Arabidopsis thaliana* [30, 33, 35, 37]. In 2009, metabolome studies were extended to diverged species such as crops and medicinal plants [53, 60, 61, 67, 68, 73, 74, 78] and to engineering studies such as quality assessment based on metabolomics [73, 74]. Thus metabolomics was applied from model species to crops and medicinal herbs. In the period of 2010-2013, metabolomics was further extended to genetics such as QTL [80, 98, 126], and to explanation of species by metabolites, i.e., ecological subjects [85] phytoalexins [119], herbivore-induced metabolites [120] and defense against pathogens [131], and to stress responses [115, 116, 127]. In addition, metabolomics has also been tried in imaging studies [112, 129]. Species-metabolite relation database KNApSAcK Core has been utilized in the extended fields of metabolomics researches and the horizon of metabolomics researches could be recognized by reviewing the works that utilized and/or cited the KNApSAcK DB.

Methodologies for multivariate analysis to statistically process the massive amount of metabolome data were reviewed in [16] and to systematize blended herbal medicines in Kampo [15]. In the following section, we focus on the mining studies of blended herbal medicines for systematically understanding the composition of medicinal herbs to efficacies on humans, that is, principal component analysis (PCA) that makes it possible to systematize the ingredient in individual blending systems, partial least squares (PLS) that can relate the ingredients of medicinal herbs to the efficacies and N-PLS that can connect multi-factors to the efficacies. We initially explain individual techniques in Section 3 and then discuss their application in data-mining of blended types of herbal medicines in Section 4.

## 3. Mathematical Methods of Data Mining

### 3.1 Principal Component Analysis (PCA)

PCA is a linear transformation of a large number of interrelated variables into a new set of variables, called as the principal components (PCs), which are uncorrelated and ordered so that the first few retain most of the variation present in all the original variables [132].

Consider a data matrix **A** = (**a**_1_ **a**_2_ … **a**_*p*_) with *n* observations and let **V** (*p* x *p*) be the variance-covariance matrix of **A**. The principal components of **A**, **Z** = (**z**_1_ **z**_2_ … **z**_*p*_), are calculated as

1 $${\text{z}}_{j}=\text{A}{\text{c}}_{\text{j}}\;(j=1,2,\ldots ,p)$$

where **c**_*j*_ is the *j*-th eigenvector of **V** which correspond to the *j*-th eigenvalue of **V** (*λ*_*j*_). The properties of PCs are: (1) Var(**z**_*j*_) = *λ*_*j*_; (2) Cov(**z**_*j*_,**z**_*j*’_) = 0, *j* ≠ *j*’; (3) Var(**z**_1_) ≥ Var(**z**_2_) ≥ … Var(**z**_p_). The cumulative proportion of variance of the original variables explained by the first *J* principal components can be obtained as

2 $$\text{Pr}(z_{j})=\frac{\sum_{j=1}^{J}\lambda_{j}}{\sum_{j=1}^{P}\lambda_{j}}$$

### 3.2 Partial Least Squares

PLSR is a regression method, which assumes underlying factors among the predictors account for most of the response variation [133, 134]. These underlying factors of *X*-variate

3 T=XW

are obtained by maximizing their covariance with the corresponding underlying factors of *Y*-variate where **X** is an *n* × *m* matrix of predictors, **Y** is an *n* × *p* matrix of responses, **T** is an *n* × *c* matrix of *X*-score factors, and **W** is *m* × *c* matrix of weight. Note that *n* is the number of observations, *m* is the number of predictors, *p* is the number of responses, and *c* is the number of components.

The *X*-score factors, i.e. matrix **T**, have the following properties [133].

a. When multiplied by loadings **P**, they are good summaries of **X**, i.e. the *X*-residuals **E** are small

4 $$\text{X}=\text{T}{\text{P}}^{\text{t}}+\text{F}$$

b. The *X*-score factors are good predictors of **Y**, i.e.

5 $$\text{Y}=\text{T}{\text{Q}}^{\text{t}}+\text{F}$$

The *Y*-residuals **F** express the deviations between the observed and modeled responses.

Based on Eq. (3), Eq. (5) can be rewritten as a multiple regression model

6 $$\text{Y}=\text{XW}{\text{Q}}^{\text{t}}+\text{F}=\text{XB}+\text{F}$$

Thus, PLSR coefficients **B** can be written as

7 $$\text{B}=\text{W}{\text{Q}}^{\text{t}}$$

whereas prediction of the responses can be obtained from

8 $$\hat{\text{Y}}=\text{XW}{\text{Q}}^{\text{t}}$$

Although PLSR is not specifically designed to discriminate among groups, Barker and Rayens [135] have demonstrated that PLSR can be used for such purposes by connecting PLSR and Linear Discriminant Analysis (LDA); this combined method is called as Partial Least Square Discriminant Analysis (PLS-DA). In PLS-DA, group membership is transformed into a dummy matrix, and this dummy matrix provides the response variables for PLSR.

### 3.3 Multiway model

**Figure 2:**
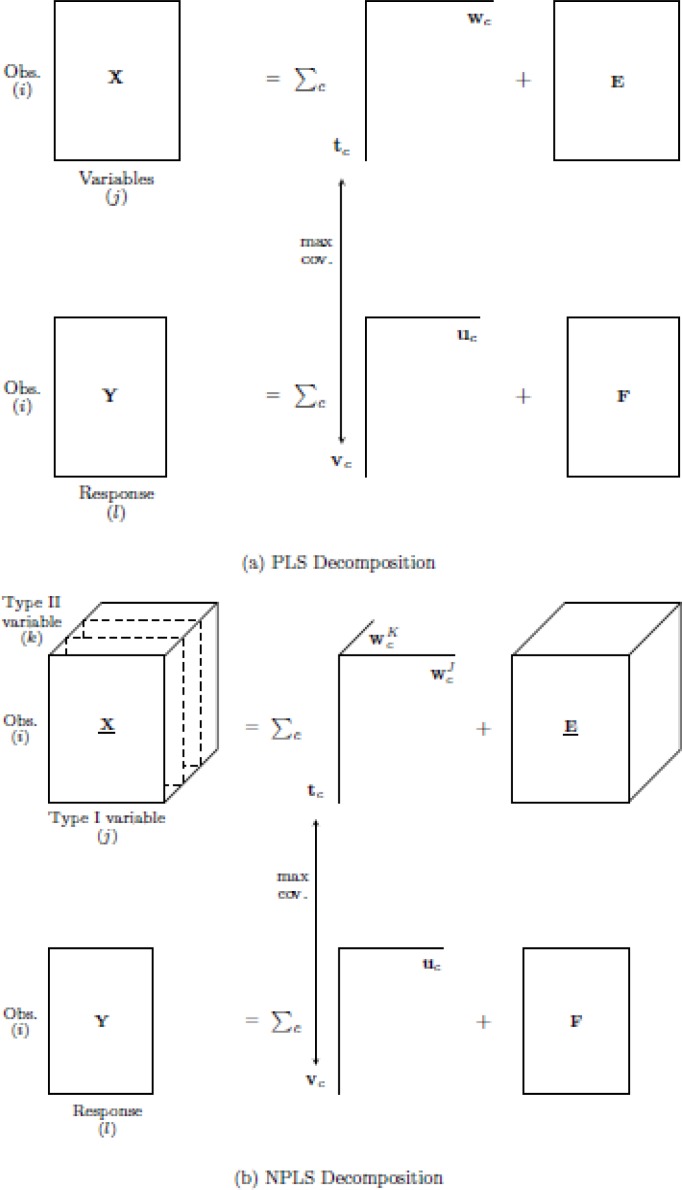
Schematic diagram of the decomposition of both predictor and response blocks for: (a) PLS and (b) N-PLS model.

An extension of PLSR to deal with multidimensional data known as Multiway Partial Least Squares has been developed by Bro [136] and is called as N-PLS. In this model, the same principle of PLSR for two dimensional data is utilized, that is, both predictor and response blocks are decomposed successively into multi-linear model such that the pairwise scores have maximal covariance. The score of the predictor is then regressed to the response variable. Fig. 2 illustrates the decomposition of N-PLS model. Moreover, N-PLS model can also be used for discrimination purpose, which is called as N-PLS-DA, that is the multiway version of PLS-DA, by utilizing the dummy matrix of group membership as the response variable.

Consider the three-dimensional array **X** indexed by observation (*i* = 1, 2, …, *I*), type I variable (*j* = 1, 2, …, *J*) and type II variable (*k* = 1, 2, …, *K*). The decomposition of both the predictor and the response block based on N-PLS model are as follows

9 $$X_{\text{ijk}}=\sum_{c=1}^{C}T_{\text{ic}}W_{\text{jc}}^{J}W_{\text{kc}}^{K}+E_{\text{ijk}}$$

10 $$Y_{\text{il}}=\sum_{c=1}^{C}V_{\text{ic}}V_{\text{lc}}+F_{\text{il}}$$

The array **X** is decomposed into a tri-linear model consisting of one score vector for observation called **t**_*c*_ (*I* x 1), and two weight vectors, one for type I variable called $W_{c}^{J}$t_c_ (I x 1), (*J* x 1) and one for type II variable called $W_{c}^{K}$(*K* x 1). Similarly, a bi-linear model is used in decomposing the matrix **Y** into one score vector **v**_*c*_ (*I* x 1) and one weight vector **u**_*c*_ (*L* x 1). The decomposition is conducted such that the covariance among the score of predictor **t** and the corresponding score of the response **v** is maximized. All scores and weights are indexed with *c* showing that they correspond to *c*th multiway component, while *C* represents the total number of multiway components used in N-PLS model. Moreover, **E** and **F** are the residuals of the decomposition of the three-dimensional array **X** and matrix **Y**, respectively.

Furthermore, let **X**_*k*_ (*I* x *J*) be the *k*th slice of **X** (*I* x *J* x *K*) for the corresponding *k*th of type II variable, then matricizing three-dimensional array **X** into matrix **X** (*I* x *JK*) is performed as follows [137]

11 $$\text{X}=[{\text{X}}_{1}][{\text{X}}_{2}]\ldots [{\text{X}}_{K}]$$

**Figure 3:**
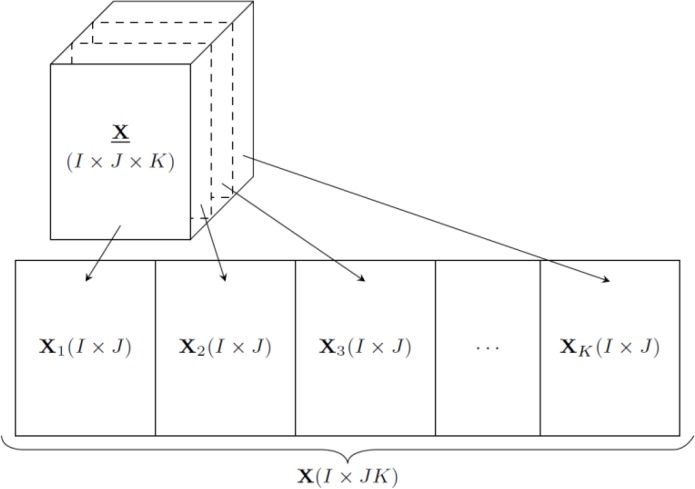
Illustration of matricizing three-dimensional array **X** (*I* x *J* x *K*) into matrix **X** (*I* x *JK*).

Fig. 3 depicts this unfolding process of array **X** into matrix **X**. Using this notation, the score **t**_*c*_ of the *c*th component can be calculated as [138]

$$t_{c}=\text{X}({\text{W}}_{c}^{K}⊗{\text{W}}_{c}^{J})$$

or

12 $$t_{\text{ic}}=\sum_{j=1}^{J}\sum_{k=1}^{K}X_{\text{ijk}}W_{\text{jc}}^{J}W_{\text{kc}}^{K}$$

From Eq. (12), the weight corresponding to *c*th component, **w**_*c*_ (*JK* x 1), can be defined as

13 $${\text{W}}_{c}=({\text{W}}_{c}^{K}⊗{\text{W}}_{c}^{J})$$

Smilde [140] also described that, due to the deflation in **X** during the decomposition, the weight matrix **W** (*JK* x *C*) can be applied directly to the original unfolded matrix **X** is defined as

14 $$\text{W}=[{\text{w}}_{2}|({\text{I}}_{\text{JK}}-{\text{w}}_{1}{\text{w}}_{1}^{t}){\text{w}}_{2}|\ldots |({\text{I}}_{\text{JK}}-{\text{w}}_{1}{\text{w}}_{1}^{t})({\text{I}}_{\text{JK}}-{\text{w}}_{2}{\text{w}}_{2}^{t})\ldots ({\text{I}}_{\text{JK}}-{\text{w}}_{Q-1}{\text{w}}_{\text{Q}-1}^{t}){\text{W}}_{Q}]$$

Hence, the scores in **T** (*I* x *C*) expressed directly in terms of the X-columns is

15 T=XW

After the decomposition procedure, the next step is to regress **Y** on the component scores **T**

16 $$\hat{\text{Y}}=\text{TB}$$

with

17 $$\text{B}={\left({\text{T}}^{t}\text{T}\right)}^{-1}{\text{T}}^{t}\text{Y}$$

From Eq. (15) and (16) we have

18 $$\hat{\text{Y}}=\text{XWB}$$

Therefore, the regression coefficients **B**_NPLS_ (*JK* x *L*) needed to predict **Y** from **X** are obtained as

19 $${\text{B}}_{\text{NPLS}}=\text{WB}$$

## 4. Illustration of Data Mining Techniques

**Figure 4:**
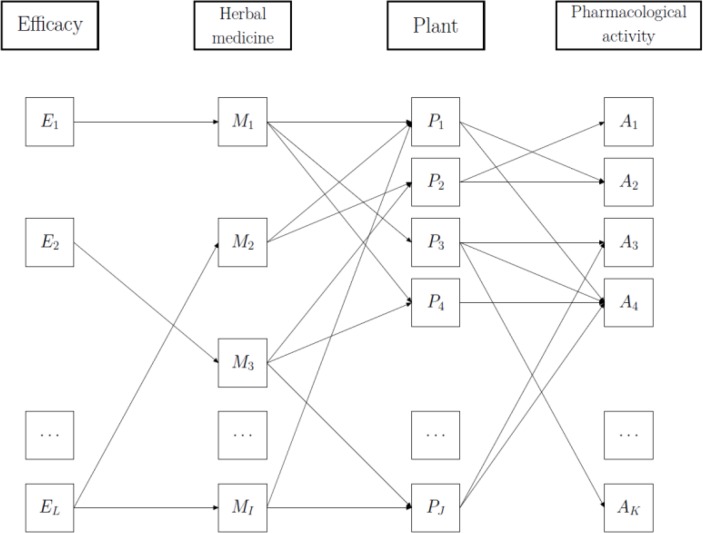
A typical network illustrating connections between efficacy, herbal medicine, plant, and pharmacological activity of plant.

Indonesia, the mega-biodiversity center like Brazil, has at least 9,600 species of plants with pharmacological activity [110] and has developed blended herbal medicines called Jamu taking modern and traditional knowledge of herbs into consideration. To prepare Jamu, several plants are selected and mixed such that the concoction has the desired efficacy. Traditionally, plants are chosen based on prior experience which is passed down from generation to generation. In curing a particular disease, each ethnic group in Indonesia may have its own formulas, whose specific nature depends strongly on the local plant resources in the region where a given population lives and the efficacies of Jamu medicines have been empirically demonstrated [139–142]. Data mining techniques with the blended herbal medicine databases such as KAMPO and JAMU (Fig. 1) makes it possible to comprehensively and mathematically understand those blended herbal systems. Fig. 4 illustrates a network connecting efficacy, herbal medicine, plant, and pharmacological activity of plant. The network showing that crude medicines M_1_, which is useful for efficacy E_1_, use three plants in its ingredients: plant P_1_, P_3_, and P_4_. Plant P_1_ has two pharmacological activities: A_2_ and A_4_. Plant P_2_ also has two pharmacological activities: A_1_ and A_2_, while plant P_3_ has three activities: A_3_, A_4_, and A_*K*_. The other connections can be described similarly.

From the concept of integrated platform of knowledge of medicinal plants and plant and human-omics depicted in Fig. 1, the efficacy layer in Fig. 4 represents the physiological activity layer in human-omics attribute, the herbal medicine and plant layer represent the prescription and medicinal herb layer, respectively, in knowledge of medicinal plants attribute, while the pharmacological activity layer represents the metabolomics layer in plant-omics attribute. On the following section we will illustrate the data mining techniques on herbal medicine database analyzing relationship among entities for two, and more than two attributes.

### 4.1 Two attributes

As an illustration for data mining of herbal medicine database which rely on relationship between two attributes, the relationship between the efficacy of Jamu and medicinal plants used in Jamu is explored using PCA [143–145]. The efficacies of 3,138 Jamu are classified into one of nine categories, namely: (1) disorders of appetite (DOA), (2) disorders of mood and behavior (DMB), (3) female reproductive organ problems (FML), (4) gastrointestinal disorders (GST), (5) musculoskeletal and connective tissue disorders (MSC), (6) pain/inflammation (PIN), (7) respiratory disease (RSP), (8) urinary related problems (URI), and (9) wounds and skin infections (WND). In total, those 3,138 Jamu use 465 plants in their ingredients. The distribution of Jamu and plant utilized in Jamu for each efficacy is shown in Table 2.

**Table 2:** Distribution of Jamu and plant utilized in Jamu for each efficacy.

Efficacy	Number of Jamu	Number of plants utilized in Jamu formulas
Urinary-related problems (URI)	72	80
Disorders of appetite (DOA)	249	148
Disorders of mood and behavior (DMB)	22	47
Gastrointestinal disorders (GST)	980	290
Female reproductive organ problems (FML)	398	182
Musculoskeletal and connective tissue disorders (MSC)	840	270
Pain and inflammation (PIN)	311	183
Respiratory diseases (RSP)	107	105
Wounds and skin infection (WND)	159	120

Note that, one plant may be used in many Jamu with varying efficacies. Hence, it is interesting to find out the most significant effects of specific plants by analyzing their usage in Jamu, and considering that the more useful a given plant in having certain effect, the more frequently the plant will be used in Jamu when that effect is desired. Biplot, a multivariate exploration tool, is suitable for this purpose because it provides simultaneous plot of principal component scores and loadings, as representation of observations and variables, respectively [145]. Considering plants as observations and efficacy groups as variables, the relationship between them can be explored using a biplot.

Following the explanation of PCA in previous section, the data matrix **A** as an input for PCA is generated by putting plant as observation and efficacy as variables. So, **A** consists of 465 rows and 9 columns. Each cell *a*_*ij*_ shows the number of Jamu that use plant *i* and useful for efficacy *j*.

**Figure 5:**
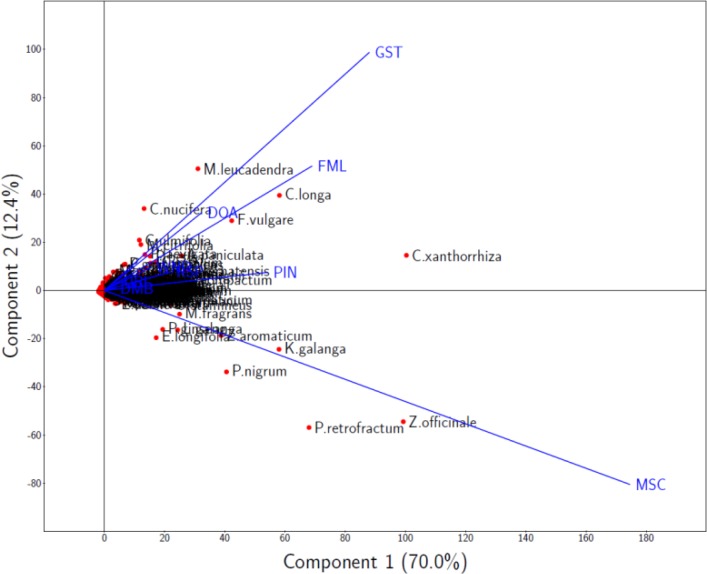
Biplot configuration based on PCA analysis of Jamu data. Plants and Jamu efficacies are represented as red points and blue lines, respectively.

**Figure 6:**
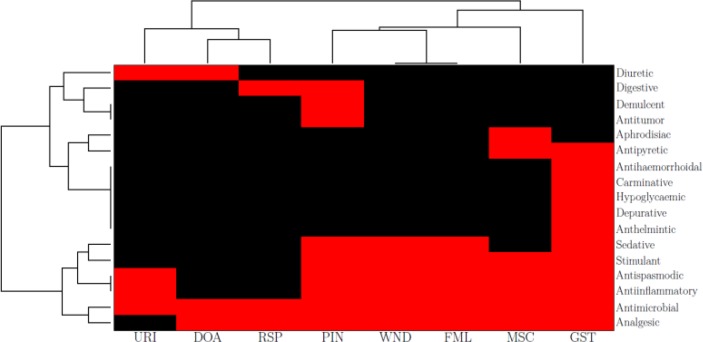
Clustergram of pharmacological activity against Jamu efficacy. The red and black cells indicate that the pharmacological activity is significant or non-significant, respectively, to the corresponding efficacy.

Biplot configuration using the first two components is shown in Fig. 5. In the figure, plants are represented as red points while Jamu efficacies as blue lines, i.e. vectors based on loadings. The length of a given efficacy line showing the variability of plant usage for the corresponding efficacy, that is, the longer the efficacy line the larger the variability of plant usage for that efficacy. From Fig. 6, it is obvious that efficacy MSC has the largest variability of plant usage, followed by efficacy GST and FML. On the other hand, efficacy DMB has the smallest variability of plant usage, followed by efficacy URI and RSP. This finding can be addressed due to two factors, that is, the number of Jamu as well as the number of plant utilized in the corresponding efficacy (see Table 2). Efficacies with large variability of plants usage (MSC, GST, and FML) have large values for both factors; in contrast, efficacies with small variability of plants usage (efficacy DMB, URI, and RSP) have small values for both factors.

In the configurations, many plants are clustered in the center. Note that, the projection value of plants’ point on a given efficacy line is the prediction of the frequency of plants usage on that efficacy. So, these clustered plants are basically plants whose frequencies of usage in Jamu are very low. In contrast to the clustered plants, some plants are spread out and located near the efficacy for which the plants are highly utilized. For example, Ginger (*Zingiber officinale*) is located near the efficacy MSC. Ginger is well known for its function of refreshing body, and for this reason many Jamu use Ginger for efficacy MSC which can easily be identified from biplot configuration. Another example is Turmeric (*Curcuma longa*) which located near the efficacy FML. Due to its analgesic and antimicrobial activity, this plant is well known and highly utilized in Indonesia as ingredient of Jamu formula for women during menstruation, which is a problem that classified into efficacy FML. Thus, the biplot configuration exhibits useful information in exploring the relationship between plants and the efficacy of Jamu.

Another illustration for relationship between two attributes on data mining of herbal medicine database is the modeling of Jamu ingredients (representation of knowledge of medicinal plants) to predict the efficacy (representation of human omics). This analysis is performed because of the fact that Jamu is prepared from a mixture of several plants. The plants are chosen so that the Jamu has the desired efficacy. As a result, the composition of the plants used in Jamu formula determines the efficacy. Thus, it is interesting to model the ingredients of Jamu, i.e. the constituent plants, and use this model to predict efficacy. PLS-DA, a statistical model for classification and discrimination based on Partial Least Square Regression (PLSR), is suitable for this analysis because a large number of plants are used in Jamu, whereas Jamu efficacies can be grouped into a few categories or classes. In this method, the plants used in each Jamu medicine served as the predictors, whereas the efficacy of each Jamu provided the responses.

The data structure used for PLS-DA is as follows. The data matrix **X** in *X*-block contains plant usage status. The dimension of matrix **X** is (*I* x *J*), where *I* is the number of Jamu (in this case, 3,138), and *J* is the number of plants (in this case, 465). Because of the availability of information about Jamu products, which generally do not state in detail the mixing ratio of the plants used, the predictors **X** is constructed only in binary data. Each cell *x*_*ij*_ (*i* = 1, 2, …, *I*; *j* = 1, 2, …, *J*) is set to 1 if Jamu *i* uses plant *j*, and is set to 0 otherwise. In the present study, nine indicator variables, which correspond to the 9 efficacies listed in Table 2 perform as the *Y*-block in PLS-DA modeling. Thus, the dimension of data matrix **Y** is (*I* x 9). Each cell *y*_*il*_ (*l* = 1, 2, …, 9) is set to 1 if Jamu *i* is classified into efficacy group *l*, and is set to 0 otherwise. Note that $\Sigma_{l=1}^{9}y_{\text{il}}=1$ because each Jamu is classified to one efficacy only.

Using the derived PLS-DA model, we can then use it to predict the efficacy of Jamu given information of the ingredients. In this analysis, among the 3,138 Jamu medicines, the efficacies of 2,248 Jamu medicines (71.6%) can be assigned to an individual efficacy reported. Hence, the efficacy in most Jamu medicines can be predicted on the basis of medicinal plants used. The percentages of correct prediction for each efficacy (see Table 3) vary from 22.7% for efficacy DMB to 89.8% for efficacy GST. The low percentage of correct prediction for efficacy DMB can be addressed due to the small number of Jamu for this efficacy, which is only 22 out of 3,138 Jamu (see Table 2).

**Table 3:** Confusion matrix of the prediction of Jamu efficacy using the PLS-DA model.

Observed efficacy	Predicted efficacy									Total	% Correct

	URI	DOA	DMB	GST	FML	MSC	PIN	RSP	WND		
URI	39	0	0	21	2	10	0	0	0	72	54.2
DOA	0	164	0	29	36	18	0	0	2	249	65.9
DMB	0	1	5	10	0	3	1	2	0	22	22.7
GST	3	17	0	880	12	46	9	6	7	980	89.8
FML	0	13	0	61	266	50	5	1	2	398	66.8
MSC	6	6	1	127	41	638	16	0	5	840	76
PIN	1	0	0	90	4	77	133	4	2	311	42.8
RSP	3	0	0	21	4	23	3	52	1	107	48.6
WND	2	3	0	57	11	11	4	0	71	159	44.7

Total	54	204	6	1296	376	876	171	65	90	3138	71.6

Furthermore, plants in the ingredients of Jamu are used as main ingredients, which contribute primarily to the medicines’ efficacies; other plants are used as supporting ingredients [146, 147]. Investigating which plants are main ingredients and which are supporting is important in order to comprehensively understand the mechanisms by which specific plants achieve desired efficacies. The regression coefficients of previous PLS-DA model, which relates plants usage in Jamu as predictors and Jamu efficacy as response, can be helpful in this attempt because they summarize the effect of plant on efficacy. Plants that act as main ingredients will have significant effect on the model developed. Furthermore, due to the absence of parametric testing for the PLS-DA coefficients, the evaluation for significance is performed using permutation testing, in which the distribution of coefficients under the null hypothesis is generated via resampling of the existing data [149].

The resampling is performed by permuting the order of the responses (in this case, Jamu efficacies) while maintaining the order of the predictors (in this case, plant utilization as Jamu ingredients) so that the existing relationship between the predictors and the response is destroyed and a new data set is generated under the null hypothesis, i.e., plant utilization in Jamu does not affect Jamu efficacy. If we perform such resampling many times and apply the PLS-DA model on the new data generated from the resampling, the accumulation of the PLS-DA coefficients obtained from this process generates a distribution, against which a *p*-value can be calculated and subsequently evaluated for significance [150].

The results of the significance testing of all plants used in each 9 efficacies are shown in Table 4. Note that one plant may be used for more than one efficacy. From the testing, we observed 234 plants (50.3% among all 465 plants) showing no significant status for all 9 efficacies; whereas the other 231 plants have significant status which comprise of 189 plants (40.6%) are significant only for 1 efficacy, 38 plants (8.2%) are significant for 2 efficacies, and the other 4 plants (0.9%) are significant for 3 efficacies. Besides testing the plants usage statistically, furthermore, we also checked from scientific papers the usage of significant plants in their corresponding efficacy. Many of the results we obtained by our analysis are supported by scientific papers.

**Table 4:** Number of significant plants for each efficacy.

Efficacy	Total		Support from scientific paper
URI	20	15	−75.00%
DOA	21	20	−95.20%
DMB	12	6	−50.00%
GST	26	23	−88.50%
FML	40	30	−75.00%
MSC	40	39	−97.50%
PIN	39	37	−94.90%
RSP	36	33	−91.70%
WND	43	38	−88.40%

Note that in predicting Jamu efficacy based on the information of its ingredients we can also use other methods such as discrimination analysis, nominal logistic regression, and support vector machine. However, in the present study we focus on PLS-DA in classifying Jamu efficacy by taking into consideration that we also intend to evaluate the significance of plant usage in Jamu to achieve specific efficacy as well as extending the analysis into three-way model by adding the plant pharmacological activity into predictors’ block.

### 4.2 More than two attributes

During the modeling process of PLS-DA in the previous section, the ingredients of Jamu provide the predictor while the Jamu efficacy serves as the response. In order to identify the function of the plants in Jamu to achieve specific efficacy, the reported pharmacological activities of the plants are added to the predictors block. Thus, the predictors block can be represented as a three-dimensional array **X** (*I* x *J* x *K*) indexed by Jamu medicine (*i*), plant (*j*), and pharmacological activity (*k*) as depicted in Fig. 2 with Jamu medicine, plant, and pharmacological activity serve as observation, type I and type II variables, respectively. Furthermore, the response block is represented as matrix **Y** (*I* x 9). This analysis then connects three attributes: (1) knowledge of medicinal plants (represented by Jamu and plants corresponding to JAMU DB in Fig. 1); (2) plant omics (represented by pharmacological activity corresponding to Biological activity (Nat) in Fig. 1); and (3) human omics (represented by efficacy).

The detail about the elements of array **X** and matrix **Y** is as the following. Let *x*_*ijk*_ (*k* = 1, 2, …, *K*; *K* = 46 where *K* is the number of reported pharmacological activity; see previous section on definition of *i*, *j*, *I*, and *J*) denotes the usage status of plant *j* with pharmacological activity *k* in Jamu *i*, where *x*_*ijk*_ = 1 if the plant *j* with pharmacological activity *k* is used in Jamu *i*, and *x*_*ijk*_ = 0 otherwise. On the other hand, let *y*_*il*_ represents the status of Jamu *i* on efficacy *l*, where *y*_*il*_ = 1 if Jamu *i* is classified into efficacy *l*, and *y*_*il*_ = 0 otherwise.

In order to identify the pharmacological activity that is significantly related with the efficacy, we adopt the guidelines from Hair et al. [150] that all weights **w** ^*K*^ (in absolute values) of 0.3 or above are significant for sample sizes of 350 or greater. Figure 6 depicts the 2-dimensional dendrogram of Jamu efficacy and the pharmacological activity significantly related with the efficacy. The cluster of Jamu efficacy and the pharmacological activity was performed using Ward Linkage based on the Euclidean distance among the entities. The clustering of the pharmacological activity side clearly exhibits two groups. The first group consists of activities useful for one or two efficacies only. This group can be regarded as a group of specific activity because the effects of the activities are specific for certain efficacy. For example the diuretic activity is useful for efficacy URI and DOA. Diuretic is an agent that increases the secretion and elimination of urine from the body [151]. Obviously, this activity is beneficial for the efficacy URI. Diuretic also help the body eliminate waste and support the whole process of inner cleansing, which is an action that is useful for efficacy DOA especially related with a slimming purpose. The five activities (antihaemorrhoidal, carminative, hypoglycaemic, depurative, and anthelmintic) are specifically related with efficacy GST. Antihaemorrhoidal means an activity that treats haemorrhoids (piles), while the carminative is defined as an activity that eases discomfort caused by flatulence. Hypoglycaemic activity helps reduce the levels of sugar in the blood, whereas the depurative eliminates toxins and purifies the system especially the blood, and the anthelmintic helpful in expelling parasites from the gut. Thus, all of these activities are helpful for the problem related with the digestive system, i.e. the efficacy GST.

Furthermore, the second group of activity revealed by the dendrogram consists of activities useful for at least four efficacies. In contrast to the first group, this group can be regarded as the general activities because of the diverse efficacies related to this group. Among all activities clustered to this group, antimicrobial activity is significantly related with all 8 efficacies. We can interpret this result as follows. Due to the environmental conditions, hygiene, and its location as a tropical country which led to many microbes that are harmful to health, then it is reasonable that antimicrobial activity is important and should be available in many Jamu formulas in Indonesia. It should be noted that many popular medicinal plants in Indonesia such as Temulawak (*Curcuma xanthorriza*), Ginger (*Zingiber officinale*), Turmeric (*Curcuma longa*) or Kencur (*Kaempferia galanga*) have content of this activity [152].

Anti-inflammation, antispasmodic, analgesic, sedative, and stimulant are also clustered into this general activity group. Since many health problems or diseases are often accompanied with inflammation or spasm, then the plants with anti-inflammation and/or antispasmodic activity are chosen in many Jamu formulas. Those health problems/diseases often cause pain or other discomforts, thus plants with certain activities such as analgesic or sedative effects are chosen in many Jamu medicines. Finally, stimulant activity, which excites or quickens activity of the physiological processes, is important for the recovery reason after one experiencing those health problems or diseases.

From the previous explanation regarding the grouping of pharmacological activity, it can be concluded that in formulating Jamu the plants are selected so that, beside curing the targeted diseases or health problems as indicated by the specific activities, the plants also should overcome the other discomforts caused by the targeted diseases or health problems as indicated by the general activities. It is in accordance with the process of making the Jamu medicines that involving whole part of plant and not only the specific active components. Hence specific or general pharmacological activities of components are involved during the curing process of Jamu medicines towards targeted diseases or health problems.

## 5. Concluding Remarks

Biology, like most scientific disciplines, is in an era of accelerated information gathering and scientists increasingly depend on the availability of amounts of data such as nucleotide and protein sequences, protein and gene expression, dynamics of metabolites etc. The nature of current systematic understanding of big data biology towards health, nutrition, and other societal issues have recently become the focus of scholar in societal studies of science and information studies. The rise of community databases, i.e., KNApSAcK family DB introduced in the present review, has been strongly associated with the current emphasis on data-intensive science. The central question is whether scientists can deduce how systems and whole organisms work from this torrent of molecular data. To progress this situation, data-intensive approach is needed for understanding intra- and inter-relations in individual layers represented in Fig. 1. The former can be solved based on a type of multivariate analyses such as cluster analysis and principal component analysis. Though the latter is more complicated, several approaches including PLS and N-PLS make it possible to clarify and understand those relations. The big data biology has become an inevitable part of biology, and the laws of nature could be clarified based on global analysis of big data biology the era of which has appeared. For centuries biological research mainly depended on experiments and for a decade or two computational analysis has usually followed experimentation but future it might be the opposite i.e., computational analysis is done first to guide the experimental design facilitated by versatile and freely available omics data at various databases.
